# Ingesting Nuts Can Regulate Adipokines Expression in Individuals Living with Overweight and Obesity: A Narrative Review of What Is Known So Far

**DOI:** 10.3390/nu17132138

**Published:** 2025-06-27

**Authors:** Stéphani Borges Campos, Mariana Buranelo Egea

**Affiliations:** 1 Faculty of Agronomy, Federal University of Goiás, Goiânia 74690-900, GO, Brazil; stephani_bc@yahoo.com.br; 2 Campus Rio Verde, Goiano Federal Institute of Education, Science, and Technology, Rio Verde 75901-970, GO, Brazil

**Keywords:** chronic inflammation, interleukins, adipose tissue, oilseeds, leukocytes

## Abstract

**Background/Objectives**: Obesity is a chronic and multifactorial disease that affects billions of people, and among the factors responsible for obesity are a sedentary lifestyle, a high-calorie diet, and genetic factors. Excessive caloric intake causes adipocyte hypertrophy and hyperplasia, contributing to the secretion of metabolically active molecules, known as adipokines, by adipose tissue. Individuals living with obesity have increased pro-inflammatory adipokines and a reduction in anti-inflammatory adipokines. Nuts contain bioactive compounds associated with potential health benefits, although these effects may vary depending on individual and dietary factors. Thus, this work aimed to critically review the impact of consuming almonds, walnuts, and mixed nuts on the production of adipokines associated with obesity and overweight. **Methods**: A comprehensive search was carried out using the terms associated with the theme of the work. The inclusion criteria for manuscripts used were the following: (1) in vivo studies; (2) intervention with oilseeds (nuts); (3) results related to adipokines and/or obesity; and (4) publications in English. **Results**: Studies show that regular intake of nuts reduces total cholesterol levels, LDL-c, and triglycerides and increases HDL-c in individuals with obesity. However, few studies demonstrate changes in adipokine levels related to the intake of nuts. A larger amount of 30 g of mixed nuts appears to be more beneficial for regulating adipokines in overweight or obese individuals than using nuts in larger amounts or isolated form. Of all the adipokines reported, only the results for IL-6 appear consistent, while the others remain unclear. **Conclusions**: Furthermore, more studies focusing specifically on this topic and humans are needed to draw greater conclusions, including the amount that results in a beneficial effect on health.

## 1. Introduction

Obesity is a chronic and multifactorial disease and has become a global epidemic with devastating consequences for health and the economy [1]. More than one billion people in the world are obese, and this number continues to rise: 650 million adults, 340 million adolescents, and 39 million children [2].

The World Health Organization defines obesity as excess body fat (body mass index, BMI, above 30 kg/m^2^), in an amount that causes harm to health [1], including long-term non-communicable conditions like insulin resistance, high blood pressure, and metabolic disorders such as cardiovascular illnesses, type 2 diabetes, persistent inflammatory conditions like rheumatoid arthritis, psoriasis, or multiple sclerosis, as well as different forms of cancer [3]. Among the factors contributing to the development of obesity, particularly in individuals with a diet high in lipids and sugar and a sedentary lifestyle, are environmental factors, as well as the influence of genetic and epigenetic factors [1].

Adipose tissue, previously considered a fat storage tissue, is currently considered a dynamic endocrine organ that secretes metabolically active molecules, such as adipokines [3]. This tissue can be divided into white adipose tissue (WAT) (present in greater quantity in the body), whose primary function is energy storage, and brown adipose tissue (BAT), responsible for thermogenesis, mainly in newborns [4].

Excessive caloric intake that results in an energy imbalance and the accumulation of adipose tissue causes hypertrophy and hyperplasia of adipocytes in individuals with obesity, leading to metabolic changes and the onset of a chronic, low-grade inflammatory state [5]. Metabolic inflammation, also known as meta-inflammation, is a response to obesity characterized by a chronic, systemic, low-intensity reaction associated with intracellular signaling pathways that trigger macrophage infiltration, endothelial cell activation, the presence of hyperplastic and hypertrophic adipocytes, and oxidative stress [6,7].

In this condition, obese individuals present an increase in pro-inflammatory biomarkers such as C-reactive protein (CRP), monocyte chemotactic protein (MCP-1), plasminogen activator inhibitor-1 (PAI-1), soluble intercellular adhesion molecule (sICAM)-1, interleukin 1 (IL-1β), tumor necrosis factor (TNF-α), interleukin 6 (IL-6), and leptin. On the other hand, adipokines with anti-inflammatory action, such as adiponectin and interleukin 10 (IL-10), are found in decreased concentrations [8,9].

Oilseeds, also known as nuts, include almonds, Brazil nuts, cashews, hazelnuts, macadamias, walnuts, pine nuts, and pistachios, among others, and are examples of foods considered by the literature as potentially functional because they contain unique chemical compositions [10], which include compounds such as proteins, dietary fiber, fatty acids, plant sterols, vitamins such as folate and tocopherols [10,11], as well as polyphenols, especially polymeric procyanidins [12]. Most of these compounds support general health [13].

Thus, the objective of this study was to conduct a critical review of the impact of consuming nuts, such as walnuts, chestnuts, and almonds, on the production of adipokines associated with obesity and overweight. For this purpose, a comprehensive search was carried out using the association of the terms “adipokines”, “obesity”, “nuts”, “almonds”, and “chestnuts” in the English language in the Scopus, PubMed, Science Direct, and Web of Science databases in July 2024. We also checked the references of the selected articles. The inclusion criteria for manuscripts used were the following: (1) in vivo study (only humans); (2) intervention with chestnuts/walnuts/almonds; (3) results related to adipokines and/or obesity; and (4) publications in English. According to the initial search strategy, 113 records were retrieved, of which 6 were excluded as duplicates and 91 as book chapters, abstracts, encyclopedias, short communications, or off-topic articles. Twelve articles were included through a secondary search (manuscripts cited in manuscripts found by systematic search but were cited by those included by systematic search). Studies that were carried out with nuts in animals, as well as studies carried out in humans with BMI < 25 kg/m^2^ were excluded. After analysis of titles and abstracts and a review of the complete text, 15 articles were included in this review.

## 2. Adipose Tissue and Its Specificities

Adipose tissue is a complex organ with innervation of the central nervous system and fundamental endocrine and immunological functions. The adipose tissue functions are energy storage, endocrine energy regulation, homeostasis, and thermoregulation [14].

Structurally, adipose tissue consists of mature adipocytes surrounded by a fraction of stromal vascular cells containing preadipocytes, macrophages, smooth muscle cells, immune cells, vessels, and a rich innervation of sensory and sympathetic fibers. Stromal vascular cells control the dynamics of adipose tissue development, homeostasis, and inflammation [15]. The extracellular matrix offers structural support and sequesters cytokines and growth factors, playing a key role in modulating both normal and pathological functions in adipose tissue [16].

Two main types of adipose tissue are observed: (i) WAT and (ii) BAT [17]. WAT, the predominant adipose tissue in the human body, stores energy in the form of hydrolyzed triacylglycerols, releasing fatty acids when the body needs energy [18]. Depending on its location, WAT is classified as visceral or subcutaneous. The first is in the omental, mesenteric, retroperitoneal, gonadal, or pericardial regions [19]. Subcutaneous is found predominantly in the superficial areas of the abdomen and back muscles and the gluteal–femoral regions. It is associated with less deleterious effects on metabolism, as it has a lower inflammatory profile [20].

**Figure 1:**
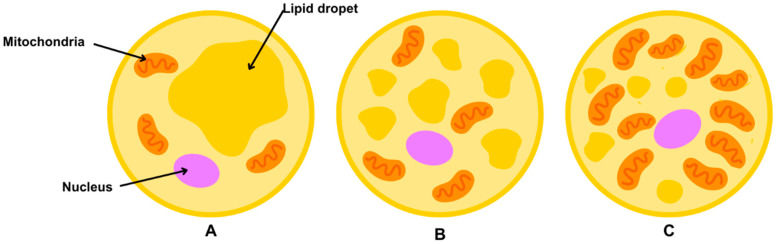
White (**A**), beige (**B**), and brown (**C**) adipocytes from adipose tissue.

White adipose tissue (Figure 1A) has an important endocrine function since it releases adipokines, which will be discussed later. Adipocytes are not the only cell type present in WAT; other cells include mesenchymal stem cells derived from adipose tissue, preadipocytes, endothelial cells, M2 macrophages, mast cells, eosinophils, dendritic cells, group 2 innate lymphoid cells, invariant natural killer T cells, and CD4^+^ regulatory T lymphocytes [21]. The proportion and profile of these cell types vary according to physiological situations [22].

Brown adipose tissue (Figure 1C) is composed mainly of brown adipocytes that derive from Myf5^+^ precursors, are characterized by numerous small lipid droplets, and are rich in mitochondria, which give them their distinctive brown coloration [23]. The nucleus is centrally located, and the endoplasmic reticulum is not well developed. Brown adipose tissue contributes to thermoregulation and is involved in heat production triggered by cold exposure or food intake [24]. After sympathetic activation, brown adipocytes release chemical energy in the form of heat. As is consistent with this, in brown adipocytes, there is a high expression of thermogenin (uncoupling protein 1, UCP1). UCP1 is a fatty acid anion/H^+^ symporter in the inner membrane of mitochondria. UCP1 dissipates the proton gradient in respiration by uncoupling cellular respiration and mitochondrial adenosine triphosphate (ATP) synthesis, thus inducing thermogenesis [25].

The secretory activity of BAT is activated by the binding of adrenaline to β3 adrenergic receptors on the surface of brown adipocytes or by prolonged exposure to cold. After prolonged activation, BAT adapts by increasing the frequency of brown adipocytes [24]. BAT activation can induce weight loss, improve insulin resistance, correct hyperlipidemia [26], protect against weight gain, and improve glucose tolerance [27].

A third type of adipose tissue concern is beige adipose tissue (Figure 1B), which is considered a transitional tissue between WAT and BAT. Beige, also known as brite adipocytes, have been detected in regions typically associated with WAT, appearing in clusters following exposure to cold stimuli [28,29]. These cells have characteristics of both brown and white adipocytes. In the basal state, they present the morphology of WAT. At the same time, after stimulation, they acquire an intermediate morphology with multiocular lipid droplets, a greater number of mitochondria, and expression markers of BAT, including UCP1 [17,29]. Different developmental lineages have been suggested for beige adipocytes depending on the tissue depot and stimuli [30,31], such as (i) transdifferentiation of mature white adipocytes [32] and (ii) maturation of brown or white preadipocytes that are present in WAT [33].

**Figure 2:**
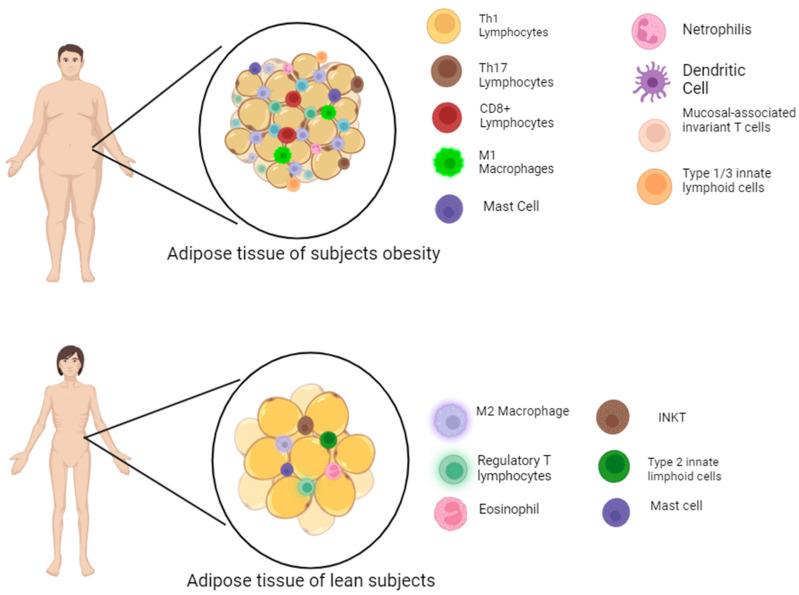
Change in cellular pattern of white adipose tissue during obesity. INKT: Invariant natural killer T.

A gradual infiltration into adipose tissue of new types of immune cells that replace resident cells is responsible for this remodeling [34]. Thus, WAT in obese individuals is infiltrated by neutrophils and pro-inflammatory immune cells, including lymphocytes (CD4^+^ Th1 and Th17 cells, CD8^+^ T cells), type 1 macrophages, γδ T cells, conventional and plasmacytoid dendritic cells, type 1 and 3 innate lymphoid cells (ILC1 and ILC3), mucosa-associated invariant T (MAIT) cells, and myeloid-derived suppressor cells (MDSCs) [21], as shown in Figure 2.

Unlike other organs, adipose tissue can also expand in adulthood, comprising more than 40% of the total body composition in patients with obesity [35]. This high plasticity is associated with a coordinated growth of the vascular system, ensuring the delivery of oxygen and nutrients to adipose tissue while facilitating the removal of metabolic waste [36]. The continuous growth, regression, and remodeling of blood vessels are regulated by metabolites and growth factors known as adipokines released by adipocytes [37].

Angiogenesis is a complex, multistep process governed by a balance of pro- and anti-angiogenic signals. This process can be triggered in response to adipocyte proliferation and increase and/or precede adipocyte proliferation and increase [38], but it is essential in modulating adipose tissue pathophysiology [39]. Adipocytes, along with other stromal cells, secrete pro- and anti-angiogenic mediators into the microenvironment to maintain vascular homeostasis and induce vascular increase or regression [37]. During BAT expansion, a transition toward an angiogenic profile supports increased vascularization and energy storage capacity [40]. Conversely, the same angiogenic phenotype in metabolically active WAT may facilitate energy consumption [41].

Furthermore, the transition from WAT to the beige phenotype is associated with an angiogenic shift with a consequent increase in vascular density. Furthermore, the vascular network serves as a supportive niche for adipocyte progenitor cells, which differentiate into preadipocytes and mature adipocytes [40,41]. Angiogenesis can be altered in pathological conditions such as obesity, metabolic syndrome, cancer, and cardiovascular pathologies [42], characterized by the abnormal expression of angiogenic factors and/or other conditions related to angiogenesis, including hypoxia, oxidative stress, hormonal imbalance, and hyperglycemia [43].

## 3. Pro- and Anti-Inflammatory Adipokines

Adipokines are peptides expressed and secreted by cells that make up adipose tissue, namely adipocytes, preadipocytes, or some cells of the immune system present in this tissue. The secretion of adipocytokines and other proteins by adipose tissue depends on factors such as (1) the volume of stored triglyceride, (2) recent whole-body energy balance and insulin/glucose signals, and (3) regulatory inputs from the nervous system and endocrine pathways, such as the hypothalamic–pituitary and growth hormone axes [44].

Adipokines act as signaling proteins that play an active role in various biological processes such as the immune response, regulation of appetite and satiety, inflammation, glucose metabolism, insulin secretion and its sensitivity at the receptor level, blood pressure, myocardial contractility, lipid accumulation in the liver, among others, in various parts of the body such as the brain, liver, pancreas, immune system cells, smooth muscle, among other tissues [45]. Adipokine production is also altered by adipose tissue hypertrophy and hyperplasia in obese individuals, supporting harmful metabolic changes that lead to insulin resistance, dyslipidemia, and increased risk of cardiovascular disease [46].

According to the effect that their activity promotes, adipokines can be classified as pro-inflammatory (a group that increases the inflammatory response and is usually present in smaller quantities) and anti-inflammatory (a group that decreases the inflammatory response and usually is present in larger quantities) [47].

The adipokines considered pro-inflammatory are leptin [48], TNF-α, interleukin-18 (IL-18) [49], resistin [50], visfatin [51], interleukin-1β (IL-1β) [52], interleukin-8 (IL-8) [53], MCP-1 [54], and CRP [55]. The adipokines considered anti-inflammatory are adiponectin, IL-10 [34], and omentin [56]. Furthermore, IL-6 may have both functions. Although it is primarily considered a pro-inflammatory cytokine involved in inflammatory and infectious responses, it also exhibits regenerative and anti-inflammatory activities [57].

**Figure 3:**
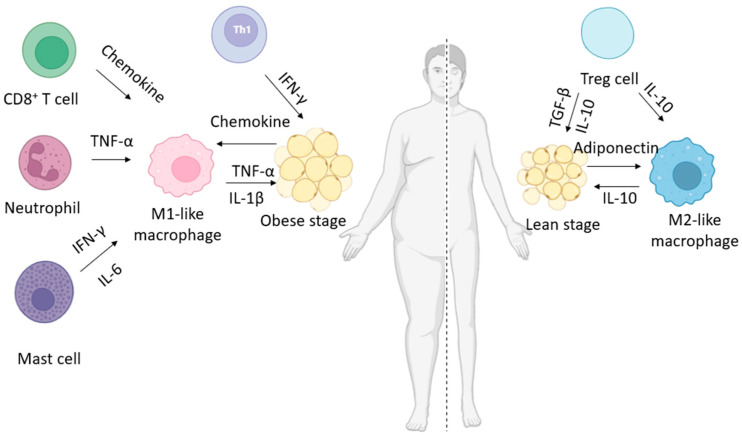
Production of adipokines due to hypertrophy and hyperplasia of adipose tissue. CD8^+^ T cell: cytotoxic T lymphocytes; IFN-γ: interferon gamma; IL-1β: interleukin 1 beta; IL-6: interleukin 6; IL-10: interleukin 10; TGF-β: transforming growth factor beta; and TNF-α: tumor necrosis factor.

Leptin concentration is proportional to the accumulation or loss of adipose tissue. In addition to being produced by adipose tissue, leptin is also expressed in other organs, such as endocrine cells of the gastrointestinal system, muscle, and brain [58]. In obese individuals, the average levels range from 30 to 50 ng/mL, while in eutrophic individuals, they range from 7 to 17 ng/mL [59]. Leptin regulates energy homeostasis and body weight by reducing hepatic glucose production glucagon levels and increasing insulin sensitivity [60] (Figure 3). Furthermore, it improves fatty acid metabolism and promotes the secretion of pro-inflammatory cytokines from macrophages and T lymphocytes while also influencing the production of oxidative and inflammatory mediators [61].

**Figure 4:**
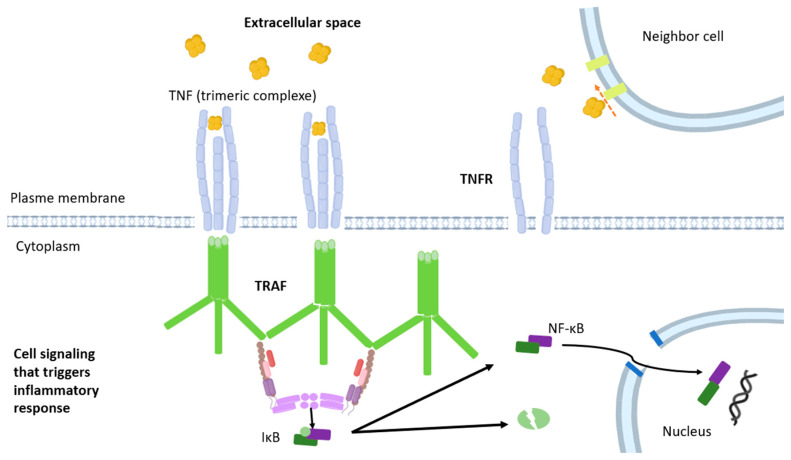
TNF exerts its functions by stimulating two different types of receptors, TNF receptor type 1 (TNFR1) and TNF receptor type 2 (TNFR2), both of which are present in macrophages. These two types of receptors activate distinct and shared signaling pathways, which may act in an interconnected manner. TRAF: tumor necrosis factor receptor-associated factor and NF-κβ: nuclear factor-kappa B.

TNF-α is composed of 233 amino acids and has a molecular weight of 25 kDa in its inactive transmembrane protein form [45]. This adipocyte activity is mediated by two receptors: TNFR1, expressed in most body cells, and TNFR2, present in immune system cells. TNF-α triggers mechanisms of apoptosis, insulin resistance, lipolysis, and regulation of insulin signaling [62] (Figure 4). In both adipose tissue and the liver, it downregulates genes associated with free fatty acid storage while upregulating those involved in cholesterol and fatty acid synthesis [63]. It also reduces the secretion of adiponectin and lipoprotein lipase, in addition to decreasing the expression of the glucose transporter 4 (GLUT4), favoring atherogenic dyslipidemia and inducing insulin resistance (Figure 3) [64].

Human resistin exists mainly in two conformations, a 660 kDa oligomer and a 45 kDa trimer, and occurs in serum concentrations ranging from 7 to 22 ng/mL [65]. This adipokine is involved in regulating glycemia lipid metabolism, controlling pituitary somatotropic cells, and modulating the hypothalamic satiety center, as well as influencing central nervous system cells. In addition, it contributes to the synthesis and secretion of pro-inflammatory cytokines and the differentiation of monocytes into macrophages. It has effects on cardiac contractility, smooth muscle cell activity, angiogenesis, endothelial permeability, renal function, and bone remodeling [66].

Apelin is a regulatory peptide derived from prepropelin, a 77-amino acid precursor processed into different active forms such as apelin-36, apelin-17, and apelin-13 [67]. Apelin expression is upregulated by insulin [68], growth hormone [69], and inflammatory factors such as TNF-α [70] and lipopolysaccharides [71]. Apelin is broadly expressed throughout the body, encompassing both peripheral tissues and the central nervous system, and plays roles in maintaining homeostasis, regulating fluid balance, supporting cell proliferation, and modulating energy metabolism [72].

Visfatin is a 52 kDa protein that shares structural identity with pre-B cell colony enhancing factor (PBEF). Subsequent studies revealed a high degree of homology between the gene encoding this protein and that of nicotinamide phosphoribosyltransferase (NAMPT) [73]. Visfatin (NAMPT), with its two isoforms, the extracellular (eNAMPT) and the intracellular (iNAMPT) form, is crucial for NAD biosynthesis, with greater activity of the extracellular form. NAD is required for several processes, including metabolic processes, glucose-stimulated insulin secretion, cell survival, cell cycle control, and apoptosis [74].

IL-18 consists of 193 amino acids and is classified as a member of the IL-1 family of cytokines based on sequence homology. IL-18 is produced as a biologically inactive precursor and is activated after cleavage by caspase-1 or other caspases [75]. IL-18, without the help of IL-12, induces Th2 cells by inducing IL-4 production from naïve T cells. IL-18 has the potential to stimulate both Th1 and Th2 responses. An increase in circulating concentrations of IL-18 may be a marker for metabolic diseases. IL-18, together with IL-12 and/or IL-15, exerts pro-inflammatory effects, such as activation of Th1 cells and induction of IFN-γ by several cells of the immune system, including NK cells. Thus, IL-18 activates components of both the innate and adaptive immune responses [76].

IL-10 is an anti-inflammatory cytokine belonging to the class II cytokine family. Its active form is a soluble homodimer of 36 kDa, consisting of two monomers with a six-α-helix structure stabilized by two intrachain disulfide bonds [77,78]. When originally described, IL-10 was classified as a cytokine explicitly secreted by T helper 2 (Th2) cells; however, it was later widely recognized that it can be produced by many myeloid and lymphoid cells [79], including CD4^+^, Th1, Th2 and Th17 cells, dendritic cells, monocytes, and macrophages [80]. The function of IL-10 is context-specific and depends on the type of cell that responds and probably also on its interaction with other inflammatory or tissue-specific signals [81]. IL-10 was initially described as functioning through signaling to macrophages and dendritic cells, thereby limiting their production of pro-inflammatory cytokines and antigen presentation capacity, which regulates Th1 responses [80]. The functional consequences of direct IL-10 signaling for specific Th cell subsets result in the preservation of FoxP3^+^ regulatory T cell activity and the inhibition of pro-inflammatory Th17 and Th2 cells (Figure 3) [80,81].

Adiponectin is a 28 kDa protein found in serum, consisting of 244 amino acids. It is characterized by four structural regions: an N-terminal signaling domain, a variable region, a collagen-like domain, and a globular domain located at the C-terminus [82]. It is produced as a single subunit that, after post-translational modifications, generates trimers (low molecular weight, LMW), hexamers (medium molecular weight, MMW), and 12–18 monomers (high molecular weight, HMW) [82]. HMW forms are more active in glycolipid metabolism [83]. Circulating adiponectin is mainly in the trimer isoform, while the 30 kDa monomeric form is found only in adipocytes (Figure 3) [82].

IL-6 is a 22–27 kDa glycosylated protein with pleiotropic effects, mainly due to its secretion by monocytes/macrophages, stromal cells, endothelial cells [34], and adipocytes, even more so during adipose tissue expansion, after lipolysis [84]. IL-6 exhibits several biological functions through two signaling pathways, which mediate its pro- and anti-inflammatory effects. These pathways are facilitated by two receptors: a membrane receptor (IL-6R) and a soluble receptor (sIL-6R) [85,86]. The expression of numerous IL-6-responsive genes, including those encoding acute phase inflammation proteins, such as C-reactive protein (CRP), is induced by the transcription factor Signal transducer and activator of transcription 3 (STAT3) modulated by the activation of Janus kinase (JAK) by IL-6 signal transduction (Figure 3) [87].

Since the presence and transduction of these adipokines depend on the individual’s conditions as well as their risk factors, which in turn depend on their health condition, diet, and physical activity, it seems clear that adipokine modulation can be achieved through diet. Thus, our emphasis is precisely on determining whether adipokine modulation can occur through the ingestion of nuts.

## 4. Impact of Nuts on Adipokine Expression in Individuals Living with Overweight and Obesity

Obesity has been associated with increased fat accumulation in adipose tissue, resulting from both adipocyte hypertrophy and hyperplasia [88]. Among the various types of body fat, visceral adipose tissue stands out as the most hormonally active, playing a central role in regulating several physiological and pathological processes [89,90]. In this context, the development of obesity is strongly associated with chronic inflammation, insulin resistance, and a series of metabolic disorders [91]. Low-grade systemic inflammation, characteristic of this condition, contributes to cardiometabolic dysfunctions and is manifested by high levels of circulating pro-inflammatory cytokines, such as IL-6, TNF-α, IL-8, leptin, IL-17, apelin, resistin, visfatin [34], IL-1β [52], IL-18 [92], and MCP-1 [54]. In contrast, there is a reduction in the production of cytokines with anti-inflammatory action, such as adiponectin, omentin, adipsin, vaspin, and IL-10, which further aggravates the inflammatory condition and its metabolic consequences [34,93].

The online bibliographic search yielded a total of 107 manuscripts, from which 7 were excluded due to being off-topic or duplicated, resulting in a total of 100 manuscripts. Based on a secondary search (in the references cited by these articles), eight additional manuscripts were included.

Table 1 presents the study design and characteristics of participants included in reviewed manuscripts that investigated the impact of nut consumption in individuals with overweight and obesity. After analyzing the abstracts, the studies were categorized into four types: comparative studies, multicenter trials, randomized controlled crossover clinical trials, and pilot studies. The studies encompass diverse populations, including patients with type 2 diabetes, metabolic syndrome, hypercholesterolemia, and high cardiovascular risk.

Mean BMI values ranged from 24.5 to 36.6 kg/m^2^, indicating that participants with varying degrees of excess weight were included. Most studies used a control group, and only a few used a placebo. The number of participants also varied widely, ranging from 15 to 511, with females predominating in several samples. The age ranges were broad, ranging from middle-aged to elderly adults, with averages ranging from approximately 40 to 67 years. These data reflect the heterogeneity of the trials and highlight the clinical relevance of investigating the effects of nuts in different metabolic contexts.

Detailed information about the research was found from the full articles published and available online and synthesized to compose Table 2 and Table S1. Thus, this narrative review included a total of 15 studies [94,95,96,97,98,99,100,101,102,103,104,105,106,107,108,109] (Table 2).

Of the total number of studies reviewed, five studies were conducted with almonds, two with walnuts, seven with mixed nuts, and one with Baru almonds. The most evaluated adipokine in the studies was IL-6 (11 studies), followed by CRP and adiponectin (7 studies), TNF-α (6 studies), MCP-1 (4 studies), IL-10 and leptin (4 studies), resistin (3 studies), IL-1b and IL-8 (2 studies), and finally, IL-18 and visfatin (only in 1 study), including in vivo models (animal and humans). Therefore, we will discuss the results found in the previously published manuscripts to understand the relationship of nuts and overweight or obesity.

### 4.1. Interleukin-6 (IL-6)

Overall, of the 11 studies with nuts that measured IL-6, 7 of them demonstrated a decrease in the concentration of this adipokine, and, evaluating separately, 2 of the 4 studies that evaluated the ingestion of 56 g of almonds demonstrated a decrease in the concentration of IL-6 [94,95]. In the two studies where there was no change in the concentration of this adipokine, the administration of almonds was carried out in a smaller quantity than in the others (42 g) [98] or for a shorter period (8 weeks) [96] (Table 2), in addition to studies that were conducted with a lower percentage of female individuals (~40%) (Table 1). In a study carried out with Baru almonds, after ingestion of 20 g, the concentration of IL-6 also decreased [108].

Unlike this pattern demonstrated by almond intake, for walnut intake, a decrease in adipokine concentration was only found when 40 g/4 weeks were administered [100]—trial composed only of female individuals (Table 1)—and there was no change when administered in greater quantities for fewer days (48 g for 4 days) [99] in studies for humans.

Mixed nut intake decreased IL-6 concentration as demonstrated by three studies, when ingested for 8 [107], 12 weeks [101], or one year [103] (30–60 g), but not when administered only for 6 weeks (30 g) [105].

**Figure 5:**
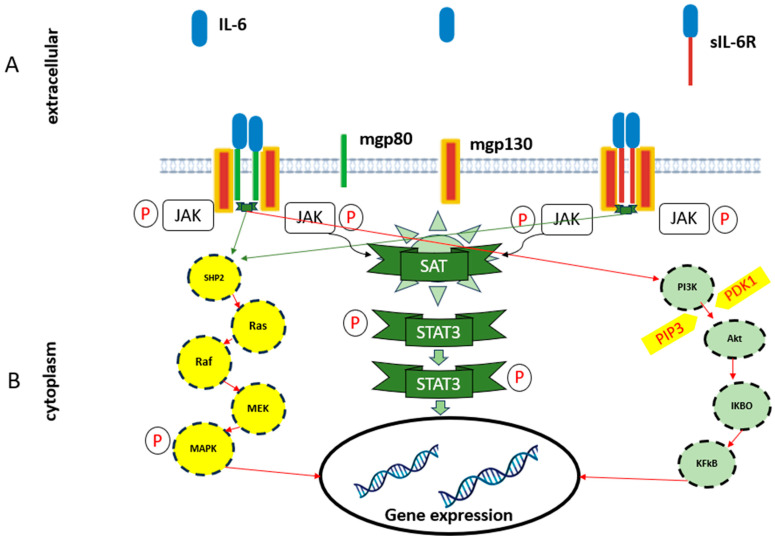
Interleukin-6 (IL-6) signaling pathways where (**A**) is the classical IL-6 pathway (membrane) considered pro-inflammatory and (**B**) is the trans-signaling pathway considered anti-inflammatory. Akt: protein kinase; IKBO: I kappa B; IL-6: interleucin-6; JAK: Janus kinase; KFkB: nuclear factor kappa-light-chain-enhancer of activated B cells; MARK: mitogen-activated protein kinase; MEK: mitogen-activated protein kinase kinase; mgp130: membrane-bound gp130; mgp80: membrane-bound gp80; P: phosphate; PDK1: 3-phosphoinositide-dependent protein kinase 1; PI3K: phosphatidylinositol 3-kinase; PIP3: phosphatidylonositrol (3,4,5)-triphosphate; Raf: RAF kinase; Ras: small GTPase; SHP2: Src homology 2-contaning protein tyrosine phosphatase2; sIL-6R: soluble interleukin-6 receptor; STAT3: signal transducer and activator of transcription 3.

IL-6 plays several biological functions, including the regulation of the immune system, hematopoiesis, metabolism, and the development of metabolic and cardiovascular diseases [110]. IL-6 pro- and anti-inflammatory biological effects occur through two signaling pathways. The classical IL-6 pathway (membrane), which occurs through the activation of the IL-6R receptor present in cells such as hepatocytes, monocytes, and macrophages, is associated with anti-inflammatory and regenerative effects [111]. Meanwhile, the trans-signaling pathway, which occurs when IL-6 binds to the soluble receptor sIL-6R and activates the gp130 protein in several cells, is related to chronic inflammatory responses, especially in individuals with obesity [57,112] (Figure 5).

During inflammatory processes, such as obesity, the excessive production of IL-6 can lead to chronic activation of the trans-signaling pathway, thereby favoring insulin resistance and contributing to the development of metabolic diseases [113]. Studies demonstrate that IL-6 secreted by WAT can induce insulin resistance through different mechanisms, including the activation of inflammatory proteins in the liver and impairment of insulin signaling in fat cells [7].

The consumption of nuts, especially almonds, appears to contribute to the reduction in IL-6 levels and, consequently, to the reduction in inflammation. This may occur because these foods are rich in bioactive compounds, such as unsaturated fatty acids, polyphenols, and phytosterols, which have anti-inflammatory properties. These compounds can modulate inflammatory signaling by reducing the activation of the IL-6 trans-signaling pathway and inhibiting the action of the transcription factor NF-κB, which is responsible for stimulating the production of inflammatory cytokines [114]. Additionally, the antioxidants present in nuts help neutralize oxidative stress, a factor that contributes to chronic inflammation [115].

Therefore, regular inclusion of almonds in the diet may be a beneficial nutritional strategy to modulate IL-6 levels, thereby reducing inflammation and its detrimental effects on metabolic health. Although, in the case of almonds and walnuts, IL-6 levels in female individuals appear to be more affected—particularly in studies composed exclusively or predominantly of women—this pattern was not observed in studies investigating mixed nuts (Table 1). Interestingly, the studies that reported a decrease in IL-6 levels following mixed nut consumption involved participants with higher average BMI (~30 kg/m^2^) compared to the study that found no change (~27 kg/m^2^). A possible explanation for this finding is that IL-6 levels are positively correlated with BMI; thus, individuals with higher BMI may exhibit elevated baseline IL-6 concentrations, allowing for a more substantial absolute reduction following dietary intervention.

### 4.2. C-Reactive Protein (CRP)

Considering all the studies reviewed, CRP measurement after nuts ingestion was performed in only six studies. Among these, a reduction in CRP was observed in one study with almond intake [94] and one with mixed nuts [107], while no significant changes were reported in other studies with almonds [96,98], walnuts [99], and mixed nuts [103,105].

IL-6 and TNF-α are mediators of CRP synthesis in the liver, especially IL-6, via activation of the STAT3 signaling pathway in hepatocytes [116]. It is plausible that reductions in IL-6 observed in some studies could be mechanistically linked to downstream changes in CRP [94]. However, this association was not consistently observed across studies, suggesting that the effect of nut consumption on CRP may be conditional.

Several mechanisms may contribute to the effects of almond consumption on CRP and IL-6 levels. Almonds are a source of magnesium, which has been inversely associated with CRP in large cohort studies [117]. Magnesium deficiency (hypomagnesemia) can intensify the inflammatory response through (i) activating phagocytic cells, (ii) activating NF-κB signaling, which in turn upregulates the transcription of some pro-inflammatory genes [118], and (iii) decreasing nitric oxide, resolvins, lipoxins, and protectins, which are anti-inflammatory markers in the body [119]. Additionally, almonds contain considerable amounts of omega-3 (ω-3) fatty acids and argininge, both of which have been linked to lower inflammatory markers including CRP concentrations [120,121]. Their content of α-tocopherol and polyphenols may also contribute to the modulation of inflammatory pathways [122,123].

Despite these proposed mechanisms, it is important to interpret the findings with caution. The only study that reported a significant reduction in CRP with almond consumption had a much smaller sample size (N = 20) compared to other studies that showed no effect [95,96,97,98], increasing the potential for type I error and limiting the generalizability of the result.

Regarding mixed nuts, in the only study that demonstrated a decrease in CRP, the research participants ingested mixed nuts to meet 20% of the calculated energy needs [107], which implies that individuals with greater body mass ingested greater absolute amounts. In addition, the observed effect may have been overestimated, since both the control group and the intervention group followed a low-calorie diet, which in itself is already known to reduce inflammatory markers.

### 4.3. Tumor Necrosis Factor (TNF-α)

In one study, a 15.7% reduction in TNF-α levels was observed in the almond diet compared to the control diet following a daily intake of 56 g/day of almonds [94] and a 14% reduction after 30 g/day of mixed nuts [103], both with over 3 months between diet types. On the other hand, no association was found between almond [96], when a shorter intake time was used (8 weeks), walnut [99,100], and mixed nut intake [102,106] and plasma TNF-α levels.

The reduction in TNF-α levels observed in the studies by Liu et al. [94] and Castaner et al. [103] can be attributed to the presence of carotenoids in these nuts. Almonds contain 68 µg/100 g (sum of lutein/zeaxanthin and β-carotene), walnuts contain 73 µg/100 g (sum of lutein/zeaxanthin and β-carotene) [124], and whole Baru almonds contain around 11.40 µg/100 g of total carotenoids [125]. These compounds possess anti-inflammatory properties, reducing oxidative stress and inhibiting the activation of NF-κB, which in turn leads to a decrease in the production of inflammatory cytokines [126].

Nuts vary considerably in the type and amount of phytochemicals, including phenolics [127], which in turn provide a variety of health benefits, including antioxidant, antitumor, and anti-inflammatory effects [128], and also appear to be correlated with benefits related to body composition [129]. Phenolic compounds present in nuts or almonds—stilbenes, tannins, lignans, phenolic acids, phenolic aldehydes, and flavonoids [127]—are inversely associated with adiposity [130].

Ellagitannins are hydrolyzable tannins detected in walnuts, pecans, and almonds and are indicated to affect mechanisms related to adiposity [131]. However, to obtain the benefits of ETs, the phenolic must first be metabolized in the gastrointestinal tract to ellagic acid through gastrointestinal tract pH changes and/or gut microbial hydrolysis via tannin hydrolase and lactonase [132]. Ellagic acid appears to inhibit adipogenesis, reduce lipogenesis, and alter adipocyte differentiation by mechanisms such as decreased expression of PPAR-γ, FA synthase (Fas), FA-binding protein 4 (aP2), and CCAAT/enhancer-binding protein α (C/EBPα) [133].

In this review, only five studies measured TNF, most likely because it is not very specific in indicating inflammation and because it can be expressed in several tissues [134]. Although the literature suggests that the inclusion of nuts and other plant foods can modulate TNF-α expression and reduce systemic inflammation, in the case of almonds and mixed nuts, it is important to highlight that the only trial conducted with a smaller number of individuals was the one that showed a significant decrease, which may be the result of experimental design bias. Thereby contributing to a better metabolic profile, the evidence remains inconclusive.

### 4.4. Monocyte Chemoattractant Protein-1 (MCP-1)

Two studies demonstrated a reduction of 11% in MCP-1 levels after intake of 30 g/day of mixed nuts during 12 weeks compared to the beginning of the study [101] and of 7% with intake of ~40 g/day of mixed nuts during 24 weeks compared to baseline [106]. On the other hand, no association was found between mixed nut [107] or walnut [100] intake and plasma MCP-1 levels. In the case of mixed nuts, this lack of effect may be associated with the shorter intake time in this study, which was 8 weeks. In addition, this study used non-salted, roasted pistachios, almonds, and peanuts as mixed nuts, unlike what happened in the other two studies [101,106].

The decrease in MCP-1, previously in studies [101,106], may be related to the fatty acid composition of mixed nuts. Wojdylo et al. [135] identified three types of fatty acids in walnuts: saturated fatty acids (SFAs) (9.2–20.3%), monounsaturated fatty acids (MUFAs) (17.5–79.3%), and polyunsaturated fatty acids (PUFAs) (7.5–69.3%). Walnuts are important sources of fatty acids, especially omega-3 and omega-6, such as linoleic acid. They also contain relevant amounts of other fatty acids, including oleic, linoleic, palmitic, and stearic acids, which have been associated with beneficial effects on serum lipids [136].

According to research carried out by López-Millán et al. [137], omega-3-PUFA supplementation is strongly correlated with a decrease in inflammatory markers in blood and adipose tissue, including IL-18, IL-6, TNF-α, ICAM-1, MCP-1, CRP, and CX3CL1. Omega-3 can regulate the activation of the NLRP3 inflammasome in adipocytes and promote the production of anti-inflammatory adipokine.

### 4.5. Visfatin, Resistin, and Leptin

There was no change in visfatin levels after ingestion of mixed nuts [97], walnut [100], and mixed nuts [104]; and leptin after ingestion of almond [97], walnut [100], and mixed nuts [104]. On the other hand, Bulló et al. [102] demonstrated a 35% increase in leptin after 30 g/day of mixed nuts for 1 year in individuals in the highest quartile of changes in glycemic index and glycemic load.

On the other hand, a study by Bulló et al. [102] demonstrated that the increase in the dietary glycemic index and glycemic load is associated with an increase in plasma leptin concentrations. Therefore, considering that higher leptin levels are related to reduced food intake or adherence to the Mediterranean diet, in addition to promoting greater energy expenditure through its action on the central hypothalamus [138], the positive regulation of leptin induced by an increase in the glycemic index or glycemic load observed in the study by Bulló et al. [102] can be considered a mechanism that favors weight loss, as was demonstrated in the same work by the decrease in the body mass index.

Furthermore, as leptin also exerts autocrine and paracrine actions in adipose tissues, it promotes lipolysis and increases fatty acid oxidation while inhibiting lipogenesis, contributing to the regulation of energy metabolism. As it is mainly known for its role in inducing satiety, higher plasma leptin levels observed after high glycemic index diets may reflect a state of leptin resistance, supporting the idea that these diets are less effective in inducing satiety than those with a low glycemic index [139].

Some bioactive compounds can modulate leptin metabolism, such as phenolic compounds [140], isothiocyanates [141], and terpenoids. Additionally, fatty acids, including docosahexaenoic acid (DHA), eicosapentaenoic acid (EPA), and oleic acid, have demonstrated antihyperlipidemic effects. The intake of PUFAs, especially EPA and DHA, appears to contribute to the reduction in circulating leptin levels, possibly through the activation of the adenosine monophosphate-activated protein kinase (AMPK) pathway [142].

### 4.6. Interleukin-8 (IL-8)

Nut intake has been associated with changes in the expression of inflammatory cytokines, including IL-8. In the study by Borkowski et al. [100], walnut consumption resulted in a 30% reduction in IL-8 levels after consumption of 40 g/day of walnuts for 4 weeks. On the other hand, Aronis et al. [99] did not demonstrate any effect on this adipokine after daily consumption of 48 g/day for 4 days. This behavior is similar to that observed for IL-6 (as cited by the authors), indicating that the modulation of these cytokines may depend on the duration of the dietary intervention.

IL-6 and IL-8, when expressed in inflamed adipocytes, show a strong negative association with LDL composition and with epoxy fatty acids derived from α-linolenic acid. However, this relationship is less evident when considering the parent acid, suggesting that the oxylipin load may be one of the mediators of the effects of LDL on inflammation [143].

In obesity, adipocytes significantly contribute to the increase in pro-inflammatory cytokines, exacerbating systemic inflammation [144]. Furthermore, lipoprotein clearance is reduced in obese individuals, resulting in an increase in low-density lipoprotein (LDL) and its oxidized form (oxLDL). Both are associated with increased secretion of inflammatory adipokines by adipose tissue [145].

LDL has been directly implicated in adipocyte inflammation, while its oxidized form has been shown to negatively correlate with plasma concentrations of adiponectin, an adipokine with anti-inflammatory properties [100].

In this context, the intake of walnuts and other nuts may modulate the expression of IL-8 and IL-6, possibly through their effects on lipid metabolism and LDL-associated inflammation. The duration of consumption appears to be a determining factor for the efficacy of this anti-inflammatory effect, suggesting that long-term dietary interventions are more effective in reducing systemic inflammation.

### 4.7. Interleukin 18 (IL-18) and Interleukin 1β (IL-1β)

Nut intake has been associated with changes in the expression of interleukins, cytokines that play a crucial role in regulating the inflammatory response. Evidence suggests that different types of nuts can modulate the production of these molecules in varying ways.

For example, one study reported that walnut consumption did not alter IL-1β levels [100], while almond intake resulted in a decrease in this adipokine [95]. Jung et al. [95] demonstrated that daily supplementation with 56 g of almonds for four weeks, within a typical Korean diet, significantly reduced serum IL-10 levels and tended to reduce IL-1β and IL-6.

The reduction in IL-1β may be related to the increased production of short-chain fatty acids (butyrate, propionate, and acetate), which are products of bacterial fermentation of dietary fiber in the intestine [146] present in 12.5 g/100 g in almonds [147]. Butyrate, in particular, has been extensively studied for its anti-inflammatory effects, including inhibition of NF-κB activation [148] and inducible nitric oxide synthase (iNOS) expression in cells in vitro [149]. Furthermore, Pedersen et al. [150] demonstrated that butyrate suppresses IL-1β-induced inflammatory gene expression and reduces nitric oxide (NO) production in mouse pancreatic islets and INS-1E cells through inhibition of the NF-κB pathway.

IL-18, in turn, was evaluated in a single study in which Casas-Agustench et al. [101] observed its reduction after the ingestion of mixed nuts. This decrease has been related to the presence of omega-3 polyunsaturated fatty acids. In a study by Troseid et al. [151], supplementation with 2.4 g/day of ω-3-PUFA also led to a reduction in IL-18 levels.

**Figure 6:**
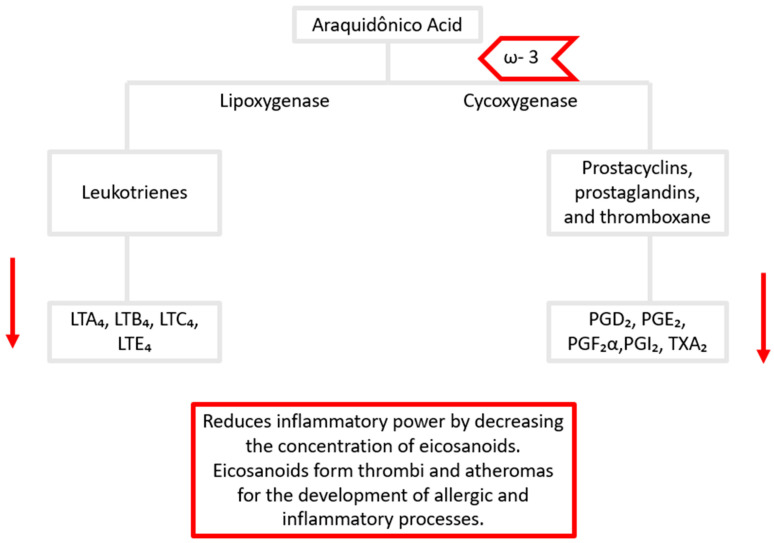
A diagram of the mechanism of arachidonic acid and eicosapentaenoic acid in the production of pro- and anti-inflammatory metabolites, respectively. ω-3: omega-3; LTA_4_: leukotriene A_4_; LTB_4_: leukotriene B_4_; LTC_4_: leukotriene C_4_; LTE_4_: leukotriene E_4_; PGD_2_: prostaglandin D_2_; PGE_2_: prostaglandin E_2_; PGF_2α_: prostaglandin F_2α_; PGI_2_: prostaglandin I_2_; TXA_2_: thromboxane A_2_. ↓ decrease.

This effect may be attributed to the partial replacement of arachidonic acid (omega-6) in the membrane phospholipids of inflammatory cells. Arachidonic acid is a precursor of series 2 prostaglandins, which are pro-inflammatory [152] (Figure 6). In contrast, EPA, a type of omega-3, serves as a substrate for the synthesis of prostaglandin E3, which has less inflammatory activity. In addition, resolvin E1, a recently identified metabolite of EPA, has demonstrated anti-inflammatory properties by inhibiting the expression of IL-8 and TNF-α [153]. These mechanisms may, in part, explain the reduction in IL-18 observed with the consumption of nuts rich in polyunsaturated fatty acids.

Therefore, the effects of nut intake on interleukin expression vary according to the type consumed and its nutritional composition. Almonds, for example, appear to reduce IL-1β, possibly through the action of butyrate. At the same time, the intake of nut-rich ω-3 may decrease IL-18 by modulating the lipid composition of cell membranes and the production of inflammatory mediators.

### 4.8. Interleukin 10 (IL-10) and Adiponectin

Studies have indicated that the consumption of almonds and Baru almonds reduces the concentration of IL-10 [95,108]. At the same time, the intake of walnuts and mixed nuts did not have a significant impact on this cytokine [106,107]. Regarding adiponectin, the intake of almonds does not appear to alter its concentration [97], while Baru almonds have been shown to increase its levels [108]. For walnuts, the results are divergent: one study demonstrated an increase [99], and in another, there was no change in the concentration of adiponectin [100]. On the other hand, for mixed nuts, two studies demonstrated an increase in adiponectin [102,104], and in one, there was no significant difference in the concentration of this marker [105].

Adiponectin is the most abundant adipokine in humans. It plays a crucial role in metabolic regulation, with its levels being reduced in conditions such as insulin resistance, glucose intolerance, dyslipidemia, and atherosclerosis [154]. Adiponectin regulation appears to be influenced by weight gain: moderate increases can elevate its levels, while significant obesity reduces its expression, mainly affecting high molecular weight (HMW) multimers [155]. Furthermore, the decline in adiponectin in obese individuals is related to factors such as hypoxia, oxidative stress, and inflammation, which impair adipose tissue homeostasis and its endocrine function [82].

The intake of walnuts, mixed nuts, and almonds has been recommended in clinical guidelines due to their nutritional composition rich in unsaturated fatty acids, fiber, antioxidants, and phytosterols [156,157]. These foods have potential benefits in reducing inflammation, improving insulin resistance, and regulating blood lipids [158,159]. Furthermore, their consumption has been associated with a reduced risk of obesity [160], hypertension [161], diabetes mellitus [162], and cardiovascular diseases [108]. Intake of 48 g/d and 40 g/d of walnuts for 4 days and 4 weeks, respectively, was associated with increased adiponectin [99] and reduced IL-6 and IL-8 [100], suggesting a beneficial effect of nuts in modulating inflammation and metabolism.

## 5. Practical Applications and Limitations

From this literature review, it seemed clear to us that, as with healthy individuals, those with chronic diseases related to overweight or obesity appear to benefit from incorporating nuts into their diet due to their chemical composition. However, it is a challenge to determine the recommended quantity.

The consumption of 20–30 g of nuts is recommended in most guidelines to achieve human health benefits in healthy individuals, such as lowering the risk of cardiovascular events (including myocardial infarction, stroke, and death from cardiovascular disease). However, globally, this recommendation for healthy individuals remains heterogeneous. For example, among the countries of the European Union, some already demonstrate that the consumption of nuts needs to be greater than 30 g, such as Ireland or Bulgaria; others provide impractical information, such as a small handful, as is the case of France; others provide quantities smaller than 20 g, such as Belgium or the Netherlands; and others still do not provide specific information and include nuts in other proteins, such as the United Kingdom [163].

This problem also affects individuals living with overweight and obesity. In this review, we found only one study that used 20 g of Baru almond in overweight or obese individuals, and this is an almond specific to South America, specifically the Cerrado region [164], and although it has some potential to aid in the treatment of this disease and its risk factors [165,166], its recommendation for inclusion in the diet is limited due to its restricted distribution (local or national).

Some clinical trials reviewed here used 30 g of mixed nuts and found beneficial effects on adipokines in individuals with overweight and obesity in longer interventions such as 3, 6, and 12 months [101,102,103,104,105]. It is essential to note that some of these results are from the PREDIMED study [101,102,103], a significant Spanish study that included individuals with high cardiovascular risk and evaluated the Mediterranean diet, yielding cumulative scientific evidence in the context of a randomized controlled trial (RCT). However, in these cases, the beneficial effect of including mixed nuts is confused with that of the Mediterranean diet, mainly if the individual uses a diet with more Western parameters (high levels of salt, sugar, or fat). In addition, these studies involve mixed nuts, and therefore, it is impossible to predict their effects and mechanisms of action on the regulation of adipokines in isolation.

Nevertheless, these studies played an important role in demonstrating that the synergy of varying dietary components (in this case, the mixed nut portion) appears to be more positive than increasing the concentration of any nuts consumed in isolation in the diet. The synergy of combining nuts in the diet still requires further confirmation and a detailed description of its potential mechanism of action in overweight and obese individuals.

Most of the other studies reviewed demonstrated that they used some nuts in isolation and carried out the test with quantities greater than 40 g; however, even with these quantities, the results seemed inadequate to us. Therefore, based on our current knowledge, it seems to us that a good strategy is to include at least 30 g of mixed nuts in the diet of individuals living with overweight and obesity, in conjunction with dietary modification and a reduction in sedentary lifestyle through the practice of physical activity, as recommended in the Mediterranean diet.

Another important limitation to report is that the studies reviewed here, although they may demonstrate future directions, remain inconclusive due to the heterogeneous groups in terms of gender and age. Another variable that needs to be isolated is the issue of diet, as some studies use low-calorie or low-fat diets, while others use the Mediterranean diet as a control.

## 6. Conclusions and Future Perspectives

The consumption of nuts, such as almonds and walnuts, has been associated with anti-inflammatory effects in some studies, particularly through the modulation of cytokine and interleukin expression involved in the inflammatory process. However, these effects are not consistently observed across all studies and may depend on factors such as the type of nut, dosage, duration of intervention, participants’ baseline inflammatory status, sex, and body composition. Studies indicate that almonds can reduce TNF-α levels, possibly due to the presence of carotenoids, which act by inhibiting the NF-κB pathway and reducing the production of pro-inflammatory cytokines. In addition, the polyunsaturated fatty acids present in nuts, especially omega-3 fatty acids, also appear to contribute to the reduction in IL-18, favoring a less aggressive inflammatory profile.

Another important factor related to the consumption of nuts is its impact on the inflammation of adipose tissue, a significant aspect of obesity, and its associated comorbidities. TNF-α and IL-6, known to play central roles in insulin resistance, can be regulated by bioactive components of nuts, such as short-chain fatty acids and antioxidant compounds. Butyrate, for example, has been associated with the inhibition of the NF-κB pathway and reduced nitric oxide production, which may attenuate the systemic inflammatory response.

Therefore, the regular inclusion of nuts in the diet may represent an effective nutritional strategy for modulating inflammation and reducing the risk of metabolic diseases. However, the effects may vary depending on the type and quantity consumed, as well as individual metabolic conditions. Further clinical studies in humans are necessary to understand these mechanisms and establish more precise nutritional guidelines for the prevention and management of inflammatory and metabolic diseases.

## Figures and Tables

**Table 1:** Study design and participant characteristics of revised manuscripts on impact of nut intake by individuals living with overweight and obesity.

References	Model	Population	BMI (kg/m^2^)	Comparison Group	N (% Female)	Age Mean
[94]	Randomized crossover controlled clinical trial	Chinese patients with type 2 diabetes mellitus	26.0 ± 0.7	Control	20 (55.0)	58.0 ± 2.0
[95]	Randomized crossover intervention trial	Korean adults with overweight or obesity *	25.4 ± 0.2	Control	84 (86.9)	52.4 ± 0.6
[96]	Randomized clinical trial	Adults with overweight or obesity	33.8 ± 5.6	Control	76 (40.8)	60.7 ± 7.7
[97]	Randomized controlled trial	Adults with obesity/adiposity considering BMI and waist circumference	27.0 ± 4.4	Control	107 (70.1)	56.2 ± 10.5
[98]	Randomized, single-blind, controlled clinical trial	Middle-aged to older individuals	29.0 ± 2.8	Control	60 (45)	61.6 ± 6.2
[99]	Randomized, double-blind, placebo-controlled crossover study	Patients with obesity and metabolic syndrome	36.6 ± 1.7	Placebo	15 (40)	58.0 ± 2.5
[100]	Randomized clinical trial	Postmenopausal hypercholesterolemic women	24.5 ± 3.5	Placebo	38 (100)	61.5 ± 5.5
[101]	Randomized parallel-group	Patients with metabolic syndrome	30.8 ± 3.1	Control	50 (40)	51.8 ± 8.4
[102]	Multicenter, controlled, randomized clinical study	Individuals with type 2 diabetes mellitus or three or more cardiovascular risk factors (current smoking, hypertension, dyslipidemia, BMI ≥25 kg/m^2^, or family history of immature cardiovascular disease	29.2 ± 0.3	Control (MD)	511 (55.6)	67.1 ± 0.4
[103]	Randomized, multicenter, parallel-group clinical trial in	Individuals at high cardiovascular risk	29.4 ± 3.7	Control (LFD)	34 (44.1)	64.3 ± 6.3
[104]	Multicenter, randomized, controlled, parallel-group clinical trial	Individuals with type 2 diabetes	29.8 ± 2.9	Control (MD)	191 (59.7)	67.2 ± 5.9
[105]	Randomized, parallel, controlled dietary intervention study with	Korean women with metabolic syndrome	27.1 ± 2.1	Control	60 (100)	35.0–65.9
[106]	Randomized controlled clinical trial	Participants with overweight/obesity	30.9 ± 0.4	Control	95 (74.7)	47.6 ± 1.8
[107]	Randomized controlled parallel-arm trial	Patients with overweight, obesity, and stable coronary artery disease	30.9 ± 3.9	Control (LCD)	67 (44.8)	58.8 ± 7.4
[108]	Randomized placebo-controlled trial	Women with overweight or obesity	33.3 ± 4.3	Placebo	46 (100)	40.0 ± 11.0

* Definition of overweight (23 ≤ BMI < 25) and obese (BMI ≥ 25) according to WHO obesity guideline for Asia–Pacific region. MD: Mediterranean diet. LFD: low-fat diet; LCD: low-calorie diet.

**Table 2:** Bibliographical survey of impact of nut intake on adipokines in individuals living with overweight and obesity. ↑ increase, ↓ decrease, and = no change.

References	Product	Dose/Study Duration	Adipokines
[94]	Almond	2 diets: control diet or almond diet (56 g/day) in a 4-week trial after a 2-week adaptation period. After the treatment period, a 2-week interval was observed between alternative diets, totaling 12 weeks.	↓ IL-6; CRP; and TNF-α.
[95]	Almond	56 g of roasted almonds or 70 g of isocaloric homemade biscuits (white flour, butter, sugar, egg, baking powder, and salt) for 4 weeks after a 2-week washout. The treatments were administered 2 weeks apart for a total of 12 weeks.	↓ IL-10; IL-1β; and IL-6.
[96]	Almond	Consumption of 56 g/day of raw almonds (28 g in the morning and 28 g in the afternoon) or consumption of 72 g/day of isocaloric sweet biscuits with a high carbohydrate content without nuts and seeds (36 g in the morning and 36 g in the afternoon) for 8 weeks.	= IL-6; TNF-α; and CRP.
[97]	Almond	2 diets: Sweet and savory mini muffins (55% available energy from carbohydrates, 36% total fat (14% saturated fat), and 10% protein) (control) or 63 g dry-roasted, unsalted whole almonds for 6 weeks.	= leptin; adiponectin; and resistin.
[98]	Almond	Supplementation with 1.5 or 3 oz (42 or 84 g, respectively) of almonds or 3.5 oz (100 g) of a snack mix containing cereal mix, coconut, dried meat, and butter for 6 months.	= IL-6 and CRP.
[99]	Walnut	Two isocaloric diets, either a placebo or a walnut diet (48 g/day), were administered for 4 days in a randomized, double-blind fashion during two different inpatient visits. The two 4-day inpatient visits were spaced 1 month apart to achieve a washout period.	↑ Adiponectin. = CRP; IL-6; IL-8; and TNF-α.
[100]	Walnut	40 g/day of walnuts for 4 weeks.	↓ IL-6 and IL-8. = adiponectin; resistin; leptin; IL-1β; and MCP-1.
[101]	Mixed nuts	Control diet and control diet supplemented with 30 g of mixed nuts (15, 7.5, and 7.5 g/day of walnuts, almonds, and hazelnuts) during the 12 weeks.	↓ IL-6; IL-18; and MCP-1.
[102]	Mixed nuts	1 L of virgin olive oil per week (for abundant use for cooking or dressing) or 30 g/day of mixed nuts (15, 7.5, and 7.5 g/day of walnuts, almonds, and hazelnuts) accompanied by a Mediterranean diet for 1 year.	↑ leptin and adiponectin ↓ IL-6 = TNF-α; resistin; visfatin; and adpsin.
[103]	Mixed nuts	15 L of olive oil for 3 months (for abundant use for cooking or dressing) or 30 g/day of mixed nuts (15, 7.5, and 7.5 g/day of walnuts, almonds, and hazelnuts) accompanied by a Mediterranean diet for 3 months.	↓ TNF-α = CRP
[104]	Mixed nuts	1 L of virgin olive oil per week (for abundant use for cooking or dressing) or 30 g/day of mixed nuts (15, 7.5, and 7.5 g/day of walnuts, almonds, and hazelnuts) accompanied by a Mediterranean diet for 1 year.	↑ Adiponectin. = leptin; visfatin; and resistin.
[105]	Mixed nuts	30 g/day of mixed nuts (15, 7.5, and 7.5 g/day of raw walnuts, raw pine nuts, and roasted peanuts, respectively) for 6 weeks.	= CRP; IL-6; and adiponectin
[106]	Mixed nut	Low-calorie diet plus a daily snack of 1.5 oz. of mixed nuts (almonds, cashews, hazelnuts, macadamia nuts, pecans, pistachios, and walnuts) (~40 g) or 1.5 oz. of pretzels during the 24 weeks.	↓ MCP-1. = IL-10 and TNF-α.
[107]	Mixed Nuts	The amount of nuts was determined based on 20% of calculated energy requirements (39 to 60 g/day) for 8 weeks. Mixed nuts contained equal amounts of non-salted roasted pistachios, almonds, and peanuts.	↓ CRP and IL-6. = IL-10 and MCP-1.
[108]	Baru Almonds	20 g/day of roasted Baru almonds for 8 weeks.	↑ Adiponectin. ↓ IL-6 and IL-10.

CRP: C-reactive protein; IL-1β: interleukin-1 beta; IL-6: interleukin-6; IL-8: interleukin-8; IL-10: interleukin-10; MCP-1: monocyte chemoattractant protein-1; TNF-α: tumor necrosis factor.

## Supplementary Materials

The following supporting information can be downloaded at: https://www.mdpi.com/article/10.3390/nu17132138/s1, Table S1: Bibliographical survey of impact of edible seed intake on biochemical and anthropometric parameters of individuals living with overweight and obesity.
