# Supplementary material for: Ingesting Nuts Can Regulate Adipokines Expression in Individuals Living with Overweight and Obesity: A Narrative Review of What Is Known So Far
Source: Nutrients. 2025 Jun 27;17(13):2138. doi: 10.3390/nu17132138 (PMC12251634; doi:10.3390/nu17132138)
Supplement: Supplementary file 1 [file nutrients-17-02138-s001.zip › nutrients-3683229-supplementary.pdf]

Supplementary table S1. Bibliographical survey of the impact of edible seed intake by individuals living with overweight and obesity on biochemical and anthropometric parameters

References	Product	Models	Dose/Study Duration	Main results
[1]	Almonds	Randomized crossover controlled clinical trial with Chinese patients with type 2 diabetes mellitus (n=20)	2 diets: control diet or almond diet (56 g/day) in a 4-week trial after a 2-week adaptation period. After the treatment period, a 2-week interval was observed between alternative diets, totaling 12 weeks	↑ latency time of Cu_{2}-induced LDL-c oxidation.<br>= Antioxidant capacity and phenolic content of plasma; plasma MDA, ICAM-1, and VCAM-1; and body weight.
[2]	Almond	Three-arm parallel randomized controlled trial in adults with BMI of 17-30 kg/m^2 (n=169)	Almonds used as a pre-meal (56 g of almonds) with 1/3 of the amount of almonds before each meal or snack (56 g of almonds) between meals during the 16-week period	↓ body fat mass; body fat; visceral fat; total cholesterol; LDL-C; and non-HDL-c.<br>= body weight and total triglycerides.
[3]	Almonds	Randomized crossover intervention trial with overweight/obese Korean adults (n=84)	56 g of roasted almonds or 70 g of isocaloric homemade biscuits (white flour, butter, sugar, egg, baking powder, and salt) for 4 weeks after a 2-week washout. The treatments were separated by 2 weeks, for a total of 12 weeks.	↑ α plasma tocoferol<br>↓ total triglycerides; TC; LDL-c; apo B; and ICAM-1.<br>= body weight; waist circumference; body composition; blood pressure; MDA; oxLDL; and plasma protein carbonyl.
[4]	Almond	Randomized clinical trial with overweight and obese adults (n=76)	Consumption of 56 g/day of raw almonds (28 g in the morning and 28 g in the afternoon) or consumption of 72 g/day of isocaloric sweet biscuits with a high carbohydrate content without nuts	↓ TC/HDL-c (women)<br>= body weight; total fat mass; fat-free mass; subcutaneous adipose tissue; visceral adipose tissue; muscle mass; waist circumference; glucose, HbA1C; insulin; ALT (serum aminotransferase); liver

			and seeds (36 g in the morning and 36 g in the afternoon) for 8 weeks	fat concentration; LDL-c; HDL-c; and total triglycerides.
[5]	Almonds	Randomized controlled trial with adults with obesity/adiposity considering BMI and waist circumference (n=107)	2 diets: Sweet and savory mini muffins (55% available energy from carbohydrates, 36% total fat (14% saturated fat), and 10% protein) (control) or 63 g dry-roasted, unsalted whole almonds for 6 weeks.	↑ plasma oleic acid and FMD.<br>↓ LDL-c and non-HDL-c.<br>= total triglycerides; HDL-c; glucose; insulin; insulin resistance; liver enzymes (fetuin-A, GGT, and ALT); BMI, waist circumference, body fat percentage, subcutaneous adipose tissue, and visceral adipose tissue.
[6]	Almond	Randomized, single-blind, controlled clinical trial with middle-aged to older individuals with BMI between 250-30 kg/m² (n=60).	Supplementation with 1.5 or 3 oz (42 or 84 g, respectively) of almonds or 3.5 oz (100 g) of a snack mix containing cereal mix, coconut, dried meat, and butter for 6 months	↑ α plasma tocoferol; visuospatial working memory; visual memory and learning; spatial planning; and working memory<br>↓ γ plasma tocopherol<br>= BMI; TC; VLDL-c; LDL-c; HDL-c; and total triglycerides; and cognitive tests.
[7]	Walnut	Randomized, double-blind, placebo-controlled crossover study in obese humans with the metabolic syndrome (n=15)	2 isocaloric diets: placebo or walnut diet (48 g/day) were administered for 4 days in a randomized, double-blind fashion during 2 different inpatient visits. The two 4-day inpatient visits were spaced 1 month apart to achieve washout.	↑ apolipoprotein A.<br>= apolipoprotein B; apoA/apoB ratio; SAA; soluble ICAM-1; ICAM-3; soluble VCAM-1; E-selectin; P-selectin; and thrombomodulin.
[8]	Walnuts	Randomized clinical trial with 38 postmenopausal hypercholesterolemic women	40g/day of walnuts for 4 weeks	↑ linoleic acid and α linoleic acid.<br>↓ postprandial cholesterol.<br>= PAI-1 and total triglycerides.

[9]	Mixed nuts	Randomized parallel group study of patients with the metabolic syndrome (n=50)	Control diet and control diet supplemented with (30 g, 15 g of walnuts + 7.5 g of almonds + 7.5 g of hazelnuts) during the 12-week period	↓ leukocytes; fasting insulin; HOMA insulin resistance; weight; waist circumference; body fat; and blood pressure.<br>= total cholesterol; total triglycerides; LDL-c; HDL-c; and glucose.
[10]	Mixed nuts	Multicenter, controlled, randomized clinical study with individuals with type 2 diabetes mellitus or three or more cardiovascular risk factors (current smoking, hypertension, dyslipidemia, BMI ≥25 kg/m² or family history of immature cardiovascular disease (n=511)	1 L of virgin olive oil per week or 30 g/day of mixed nuts (30 g, 15 g of walnuts + 7.5 g of almonds + 7.5 g of hazelnuts) accompanied by a Mediterranean diet for 1 year.	↓ BMI.<br>= waist circumference.
[11]	Mixed nuts	Randomized, multicenter, parallel-group clinical trial in individuals at high cardiovascular risk (n = 34)	15 L of olive oil or or 30 g/day of mixed nuts (15 g/d of walnuts+ 7.5 g/d hazelnut + 7.5 g/d almond) for 3 months	↑ plasma α-linolenic acid<br>↓ ICAM-1; IGFR2; and PTGS2<br>= oxLDL
[12]	MIxed nuts	Multicenter, randomized, controlled, parallel-group clinical trial (n=191) with individuals with type 2 diabetes	1 L of virgin olive oil per week or 30 g/day of mixed nuts (30 g, 15 g of walnuts + 7.5 g of almonds + 7.5 g of hazelnuts) accompanied by a Mediterranean diet for 1 year.	↑ adiponectin/HOMA-IR.<br>= BMI; waist circumference; blood pressure; and HOMA-IR.
[13]	MIxed nuts	Randomized, parallel, controlled dietary intervention study with Korean women with metabolic syndrome (n=60)	2 diets: control diet and treatment diet (the same control diet supplemented with 30 g/day of mixed nuts (15, 7.5 and 7.5 g/day of raw walnuts, raw pine nuts and roasted peanuts respectively) for 6 weeks.	↓ total cholesterol and non-HDL-c (women).<br>= BMI; weight; and waist circumference.

[14]	Mixed nut	Randomized controlled clinical trial with 95 participants.	Low-calorie diet plus a daily snack of 1.5 oz. of mixed nuts (almonds, cashews, hazelnuts, macadamia nuts, pecans, pistachios, and walnuts) (~40 g) or 1.5 oz. of Pretzel during the 24-week period.	= TC; total triglycerides; and HDL-c.<br>↓ body weight; BMI; diastolic pressure; and heart rate.
[15]	Mixed Nuts	Randomized controlled parallel-arm trial with overweight and obese stable coronary artery disease patients (n=67)	The amount of nuts was determined based on 20% of calculated energy requirements (39 to 60 g/day) for 8 weeks. Mixed nuts contained equal amounts of non-salted roasted pistachios, almonds, and peanuts.	↓ ICAM-1; body weight; and waist circumference
[16]	Baru Almonds	Randomized placebo-controlled trial with Overweight and Obese Women (n=46)	20 g/day of roasted baru almonds for 8 weeks.	↑ glutathione peroxidase activity; Superoxide dismutase activity; and copper.<br>↓ BMI; waist circumference; and cobalt.<br>= zinc, calcium, iron, phosphorus, magnesium, manganese, selenium and strontium.

ALT: alanine transaminase; apo B: Apolipoprotein B; BMI: body mass index; CRP: C-reactive protein; HbA1C: hemoglobin A1C; HDL-c: High-density lipoprotein cholesterol; HOMA-IR: Homeostatic Model Assessment for Insulin Resistance; FMD: flow mediated dilation; GGT: γ-glutamyltransferase; ICAM-1: Intercellular Adhesion Molecule 1; IGFR2: Insulin-like growth factor 2 receptor; IL-1β: Interleukin-1 beta; IL-6: Interleukin-6; IL-8: Interleukin-8; IL-10: Interleukin-10; LDL-c: Low-density lipoprotein cholesterol; MCP-1: Monocyte chemoattractant protein-1; MDA: Malondialdehyde; oxLDL: Oxidized Low-density Lipoprotein; PAI-1: Plasminogen activator inhibitor-1; PTGS2: prostaglandin-endoperoxide synthase 2 or cyclooxygenase 2; TC: total cholesterol; TNF-α: Tumor necrosis factor; VCAM-1: vascular cell adhesion molecule 1; and VLDL-c: Very low-density lipoprotein cholesterol

1. Liu, J.-F.; Liu, Y.-H.; Chen, C.-M.; Chang, W.-H.; Chen, C.O. The effect of almonds on inflammation and oxidative stress in Chinese patients with type 2 diabetes mellitus: a randomized crossover controlled feeding trial. *European journal of nutrition* **2013**, *52*, 927-935.

2. Liu, Y.; Hwang, H.-J.; Ryu, H.; Lee, Y.-S.; Kim, H.-S.; Park, H. The effects of daily intake timing of almond on the body composition and blood lipid profile of healthy adults. *Nutrition Research and Practice* **2017**, *11*, 479-486.
3. Jung, H.; Chen, C.-Y.O.; Blumberg, J.B.; Kwak, H.-K. The effect of almonds on vitamin E status and cardiovascular risk factors in Korean adults: a randomized clinical trial. *European journal of nutrition* **2018**, *57*, 2069-2079.
4. Bowen, J.; Luscombe-Marsh, N.D.; Stonehouse, W.; Tran, C.; Rogers, G.B.; Johnson, N.; Thompson, C.H.; Brinkworth, G.D. Effects of almond consumption on metabolic function and liver fat in overweight and obese adults with elevated fasting blood glucose: A randomised controlled trial. *Clinical nutrition ESPEN* **2019**, *30*, 10-18.
5. Dikariyanto, V.; Smith, L.; Francis, L.; Robertson, M.; Kusaslan, E.; O'Callaghan-Latham, M.; Palanche, C.; D'Annibale, M.; Christodoulou, D.; Basty, N. Snacking on whole almonds for 6 weeks improves endothelial function and lowers LDL cholesterol but does not affect liver fat and other cardiometabolic risk factors in healthy adults: The ATTIS study, a randomized controlled trial. *The American journal of clinical nutrition* **2020**, *111*, 1178-1189.
6. Mustra Rakic, J.; Tanprasertsuk, J.; Scott, T.M.; Rasmussen, H.M.; Mohn, E.S.; Chen, C.-Y.O.; Johnson, E.J. Effects of daily almond consumption for six months on cognitive measures in healthy middle-aged to older adults: a randomized control trial. *Nutritional Neuroscience* **2022**, *25*, 1466-1476.
7. Aronis, K.N.; Vamvini, M.T.; Chamberland, J.P.; Sweeney, L.L.; Brennan, A.M.; Magkos, F.; Mantzoros, C.S. Short-term walnut consumption increases circulating total adiponectin and apolipoprotein A concentrations, but does not affect markers of inflammation or vascular injury in obese humans with the metabolic syndrome: data from a double-blinded, randomized, placebo-controlled study. *Metabolism* **2012**, *61*, 577-582.
8. Borkowski, K.; Yim, S.J.; Holt, R.R.; Hackman, R.M.; Keen, C.L.; Newman, J.W.; Shearer, G.C. Walnuts change lipoprotein composition suppressing TNFα-stimulated cytokine production by diabetic adipocyte. *The Journal of nutritional biochemistry* **2019**, *68*, 51-58.
9. Casas-Agustench, P.; López-Uriarte, P.; Bulló, M.; Ros, E.; Cabré-Vila, J.; Salas-Salvadó, J. Effects of one serving of mixed nuts on serum lipids, insulin resistance and inflammatory markers in patients with the metabolic syndrome. *Nutrition, metabolism and cardiovascular diseases* **2011**, *21*, 126-135.
10. Bulló, M.; Casas, R.; Portillo, M.; Basora, J.; Estruch, R.; Garcia-Arellano, A.; Lasa, A.; Juanola-Falgarona, M.; Arós, F.; Salas-Salvadó, J. Dietary glycemic index/load and peripheral adipokines and inflammatory markers in elderly subjects at high cardiovascular risk. *Nutrition, metabolism and cardiovascular diseases* **2013**, *23*, 443-450.
11. Castaner, O.; Corella, D.; Covas, M.-I.; Sorlí, J.V.; Subirana, I.; Flores-Mateo, G.; Nonell, L.; Bullo, M.; de la Torre, R.; Portoles, O. In vivo transcriptomic profile after a Mediterranean diet in high–cardiovascular risk patients: A randomized controlled trial. *The American journal of clinical nutrition* **2013**, *98*, 845-853.
12. Lasa, A.; Miranda, J.; Bulló, M.; Casas, R.; Salas-Salvadó, J.; Larretxi, I.; Estruch, R.; Ruiz-Gutiérrez, V.; Portillo, M.P. Comparative effect of two Mediterranean diets versus a low-fat diet on glycaemic control in individuals with type 2 diabetes. *European journal of clinical nutrition* **2014**, *68*, 767-772.
13. Lee, Y.J.; Nam, G.E.; Seo, J.A.; Yoon, T.; Seo, I.; Lee, J.H.; Im, D.; Bahn, K.-N.; Jeong, S.A.; Kang, T.S. Nut consumption has favorable effects on lipid profiles of Korean women with metabolic syndrome. *Nutrition Research* **2014**, *34*, 814-820.
14. Wang, J.; Wang, S.; Henning, S.M.; Qin, T.; Pan, Y.; Yang, J.; Huang, J.; Tseng, C.-H.; Heber, D.; Li, Z. Mixed tree nut snacks compared to refined carbohydrate snacks resulted in weight loss and increased satiety during both weight loss and weight maintenance: a 24-week randomized controlled trial. *Nutrients* **2021**, *13*, 1512.

15. Ghanavati, M.; Hosseinabadi, S.M.; Parsa, S.A.; Safi, M.; Emamat, H.; Nasrollahzadeh, J. Effect of a nut-enriched low-calorie diet on body weight and selected markers of inflammation in overweight and obese stable coronary artery disease patients: a randomized controlled study. *European journal of clinical nutrition* **2021**, *75*, 1099-1108.
16. de Souza, R.G.M.; Gomes, A.C.; Navarro, A.M.; Cunha, L.C.d.; Silva, M.A.C.; Junior, F.B.; Mota, J.F. Baru almonds increase the activity of glutathione peroxidase in overweight and obese women: a randomized, placebo-controlled trial. *Nutrients* **2019**, *11*, 1750.
